# Technical Modifications to the Y-Incision Aortic Annular Enlargement Technique

**DOI:** 10.1055/a-2836-2224

**Published:** 2026-03-26

**Authors:** Amine Fikani, Victor Jebara

**Affiliations:** University Medical Center Hotel-Dieu de France Hospital, Faculty of Medicine, Saint-Joseph University of Beirut, Beirut, Lebanon

**Keywords:** aortic valve and root, heart valve surgery, cardiac

## Abstract

Aortic annular enlargement has become an essential component of contemporary aortic valve surgery, particularly in the context of patient–prosthesis mismatch. We describe several practical technical adaptations to a Y-incision–based annular enlargement approach, derived from our institutional experience. These modifications focus on patch material selection, suturing strategy, and external reinforcement, with the aim of simplifying execution and enhancing hemostatic reliability while preserving the conceptual framework of the technique.

Aortic annular enlargement (AAE) techniques play a critical role in contemporary aortic valve surgery by enabling implantation of adequately sized prostheses and reducing the risk of patient–prosthesis mismatch. Among the various approaches described, the Y-incision AAE technique with a rectangular Dacron patch provides an elegant and reproducible approach for achieving substantial upsizing of the aortic root while maintaining excellent safety outcomes. 1 2 In our practice, we have adopted the general principles of the Y-incision AAE, but we have introduced some technical modifications that we believe further simplify the procedure and improve the quality of the repair ( Fig. 1 ).

**Fig. 1:**
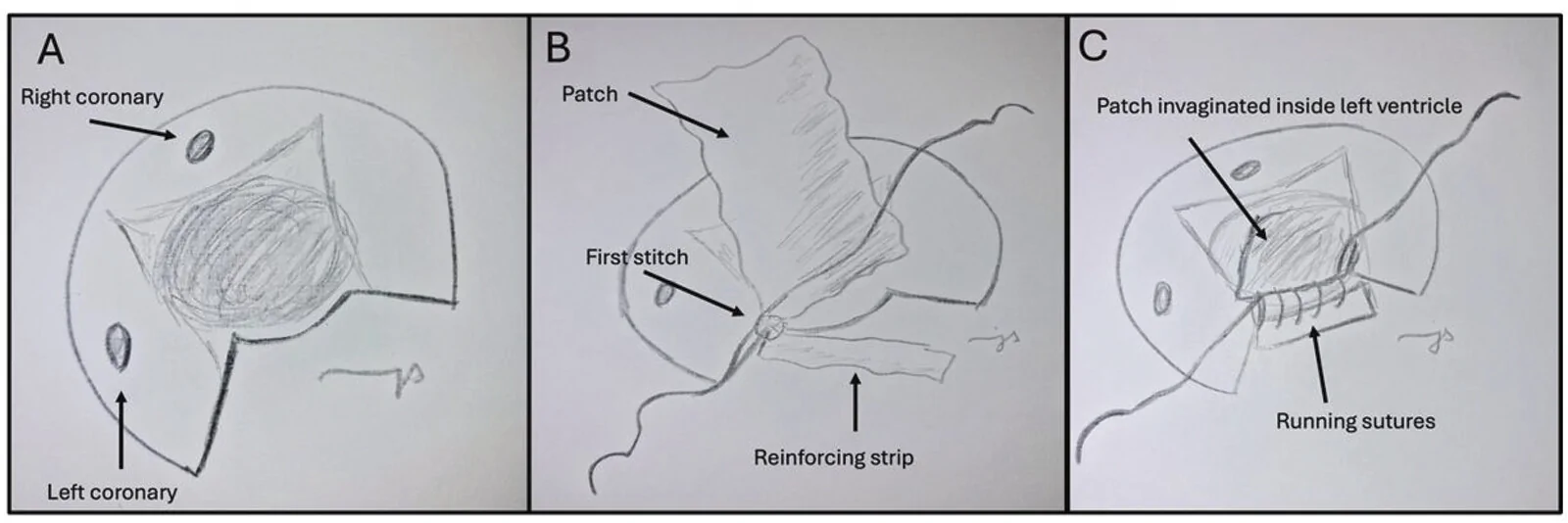
( **A** ) Classic Yang incision. ( **B** ) First anchoring stitch between the patch, the annulus, and the pericardial reinforcing strip. ( **C** ) The patch is invaginated inside the left ventricle. Running sutures along the annulus. Source: Original illustration © Manal Jean Sroujy.

First, instead of using a Dacron patch, we routinely employ a bovine pericardial patch. We have found bovine pericardium to be more pliable and easier to suture.

Second, we have modified the suturing of the patch. After placing the first anchoring stitch at the level of the left trigone, we tie this suture down, thus securing the proximal point of fixation. We then invaginate the pericardial patch inside the ventricle, which provides excellent exposure of the suture line and facilitates precise alignment between the patch and the annulus. Once the patch is positioned intraventricularly, we complete the running suture toward the right trigone.

Third, during the suturing of the patch to the annulus, we routinely place a reinforcing strip of bovine pericardium externally, running along the outside of the aortic root. This reinforcement provides additional hemostatic security in this region where bleeding can be difficult to control once the prosthesis is seated. In our experience, this step has significantly reduced the need for additional hemostatic maneuvers after unclamping.

These technical adaptations preserve the fundamental principles of Y-incision–based aortic annular enlargement while aiming to simplify patch handling, improve exposure during suturing, and enhance hemostatic security at a critical step of the operation. In our experience, these refinements have facilitated execution of the procedure and increased confidence in the durability of the repair. We believe that sharing such practical modifications may contribute to the ongoing evolution of AAE techniques and support broader adoption of effective strategies for annular enlargement in aortic valve surgery.
